# A novel cuproptosis-related prognostic lncRNA signature and lncRNA MIR31HG/miR-193a-3p/TNFRSF21 regulatory axis in lung adenocarcinoma

**DOI:** 10.3389/fonc.2022.927706

**Published:** 2022-07-22

**Authors:** Xiaocong Mo, Di Hu, Pingshan Yang, Yin Li, Shoaib Bashir, Aitao Nai, Feng Ma, Guoxia Jia, Meng Xu

**Affiliations:** ^1^ Department of Oncology, The First Affiliated Hospital of Jinan University, Jinan University, Guangzhou, China; ^2^ Department of Neurology and Stroke Centre, The First Affiliated Hospital of Jinan University, Guangzhou, China; ^3^ Department of Thoracic Surgery, The First Affiliated Hospital of Jinan University, Guangzhou, China

**Keywords:** lung adenocarcinoma, cuproptosis, MIR31HG, miR-193a-3p, TNFRSF21

## Abstract

Lung adenocarcinoma (LUAD) remains the most common subtype of lung malignancy. Cuproptosis is a newly identified cell death which could regulate tumor cell proliferation and progression. Long non-coding RNAs (lncRNAs) are key molecules and potential biomarkers for diagnosing and treating various diseases. However, the effects of cuproptosis-related lncRNAs on LUAD are still unclear. In our study, 7 cuproptosis-related lncRNAs were selected to establish a prognostic model using univariate Cox regression analysis, LASSO algorithm, and multivariate analysis. Furthermore, we evaluated AC008764.2, AL022323.1, ELN-AS1, and LINC00578, which were identified as protective lncRNAs, while AL031667.3, AL606489.1, and MIR31HG were identified as risk lncRNAs. The risk score calculated by the prognostic model proved to be an effective independent factor compared with other clinical features by Cox regression analyses [univariate analysis: hazard ratio (HR) = 1.065, 95% confidence interval (CI) = 1.043–1.087, *P* < 0.001; multivariate analysis: HR = 1.067, 95% CI = 1.044–1.091, *P* < 0.001]. In addition, both analyses (ROC and nomogram) were used to corroborate the accuracy and reliability of this signature. The correlation between cuproptosis-related lncRNAs and immune microenvironment was elucidated, where 7 immune cells and 8 immune-correlated pathways were found to be differentially expressed between two risk groups. Furthermore, our results also identified and verified the ceRNA of cuproptosis-related lncRNA MIR31HG/miR-193a-3p/TNFRSF21 regulatory axis using bioinformatics tools. MIR31HG was highly expressed in LUAD specimens and some LUAD cell lines. Inhibition of MIR31HG clearly reduced the proliferation, migration, and invasion of the LUAD cells. MIR31HG showed oncogenic features *via* sponging miR-193a-3p and tended to positively regulate TNFRSF21 expression. In a word, lncRNA MIR31HG acts as an oncogene in LUAD by targeting miR-193a-3p to modulate TNFRSF21, which may be beneficial to the gene therapy of LUAD.

## Introduction

Lung adenocarcinoma (LUAD) is one of the most common subtypes of lung malignancy, and it ranges from initially non-invasive tumors to high-mortality-specific invasive tumors (1, 2). Although a number of biomarkers or diagnostic tools that may be employed to predict the prognosis of LUAD have been discovered in recent years (3, 4), they are still in the stage of molecular research (5). Therefore, it will be indispensable to identify promising biomarkers and prognostic models to reveal the prognostic genetic characteristics of LUAD and obtain the most accurate clinical information.

Cuproptosis is a novel mode of cell death that is relevant to copper and mitochondrial respiration. The pathological mechanism is that copper interacts directly with the fatty acylated components of the tricarboxylic acid (TCA) cycle, leading to excessive aggregation of fatty acylated proteins and loss of iron–sulfur cluster proteins, which stimulates proteotoxic stress and cell death (6). Recent studies have found a close relationship between copper cell death and human cancer (7, 8), which proved that cuproptosis is closely related to the development of cancers, but it remains unclear in LUAD.

lncRNA is a newly discovered functional lncRNA which has the ability to mediate various mechanisms through their multiple functions and plays vital roles in a large number of cancer processes (9, 10). Recent studies have confirmed that lncRNAs regulated the early development of LUAD through different signaling pathways—for example, lncRNA JPX promoted the tumorigenesis in LUAD by sponging miR-33a-5p (11), and lncRNA GMDS-AS1 could inhibit LUAD *via* regulating the miR-96-5p/CYLD pathway (12). Besides this, lncRNA FAM83A-AS1 accelerated LUAD migration, proliferation, and invasion (13). However, the precise involvement of cuproptosis-related lncRNAs in LUAD is still ambiguous.

Our current work was designed to delve deeper into the expression profiles of cuproptosis-related lncRNAs and their relationship with the immune microenvironment as well as validate potentially relevant regulatory mechanisms in LUAD. Our findings would provide valuable references for efficient prognostic biomarkers and the diagnosis of LUAD.

## Materials and methods

### Data collection and processing

The data were all obtained from The Cancer Genome Atlas (TCGA; https://portal.gdc.cancer.gov/). The LUAD microarray gene profiling dataset in this research was obtained from the GEO NCBI web server (https://www.ncbi.nlm.nih.gov/geo/). Patients with missing survival information were excluded. Perl (https://www.perl.org, version 5.32.1) was used to collate the clinical details.

### Identification of cuproptosis-related genes

Cuproptosis-correlated genes were identified based on previous reports (6, 14, 15). In total, 39 mRNAs were finally selected as differentially expressed genes (DEGs) by the packages of “limma” and “pheatmap”. Then, a PPI network (related to protein–protein interaction) of the 39 DEGs was established by a search tool to retrieve interacting genes (STRING, https://string-db.org/).

### KEGG and GO enrichment analysis

The Gene Ontology (GO; http://www.geneontology.org/) and the Kyoto Encyclopedia of Genes and Genomes (KEGG; http://www.genome.jp/kegg/) enrichment analyses were conducted using the “ggplot2” R package. The GO database was performed to analyze the biological characteristics of these cuproptosis-related genes. KEGG was performed to detect the signaling pathway of cuproptosis-related genes.

### Identification of cuproptosis-related lncRNAs and prognosis model construction

LcRNAs related to cuproptosis-related DEGs were screened out based on Pearson correlation analysis. We randomly classified the included cases (*n* = 504) into training and validation cohorts at a 1:1 ratio. Cuproptosis-related lncRNAs were selected following univariate Cox regression analysis, LASSO Cox algorithm, and multivariate analysis. These cuproptosis-related lncRNAs were chosen to establish a prognostic model (the risk score = expression_lncRNA1_ × coefficient _lncRNA1_ + expression_lncRNA2_ × coefficient _lncRNA2_ + … + expression_lncRNAn_ × coefficient _lncRNAn_). We then analyzed the hazard ratio (HR) of prognostic factors for distinguishing between protective lncRNA (HR >1) and risk lncRNA (HR <1). These cuproptosis-related lncRNAs were further visualized *via* Cytoscape and Sankey diagram. Furthermore, the patients were classified into two risk groups (high and low).

### Clinical meaning of the prognostic model

Univariate and multivariate Cox regressions were performed to identify whether the risk score and clinical features (age, gender, grade, *etc*.) were valuable prognostic indicators for LUAD patients. Nomograms were used to show the clinical features and risk scores of survival rates.

### Competing endogenous RNA network construction

To further elucidate the potential mechanism of cuproptosis-related lncRNAs in LUAD, we constructed a ceRNA network. Mircode (www.mircode.org) was utilized to predict the miRNA targets connecting to cuproptosis-related lncRNAs. After miRNA identification, TargetScan (http://www.targetscan.org/vert_72/) and miRDB databases (http://mirdb.org/) were utilized to predict mRNA targets interacting with miRNAs.

### Cell culture and clinical specimens

The LUAD cell lines (A549, NCI-H2009, and PC9) and bronchial epithelioid cells (HBE) were generous gifts from Dr. Feng Ma. The LUAD cells were cultured in RPMI-1640 medium (Hyclone; GE Healthcare) and maintained in a humidified incubator at 37°C in 5% CO_2_. In total, 34 paired LUAD tissues (T) and normal specimens (N) were collected from the First Affiliated Hospital of Jinan University from April 2020 to May 2021. Our research concerning human tissues was reviewed and approved by the Ethics Committee of The First Affiliated Hospital of Jinan University. All patients signed informed consents in the present study.

### Cell transfection

si-MIR31HG, miR-193a-3p inhibitor, miR-193a-3p mimics, and their negative control (si-ctrl) were synthesized by GenePharma (Shanghai, China). NCI-H2009 and A549 cells were evenly plated in 96-well plates. When the two cells reached about 80–90% confluence, they were transfected with plasmid by Lipo3000 (Invitrogen, Carlsbad, CA, USA). NCI-H2009 and A549 cells were harvested for the subsequent experiments following incubation at 37°C for 48 h.

### Cell proliferation assay

96 −well plates were taken to seed LUAD cells (2 × 10^5^ cells/well) (A549 and NCI-H2009). After incubation at 37°C and 5% CO_2_ for various times, a CCK−8 reagent test kit, which was provided from Tiangen (Hangzhou, China), was mixed at 10 µl/well, and LUAD cells were evenly incubated for 3 h at 37°C and 5% CO_2_. Finally, we read the absorbance at 450 nm on the enzyme labeling instrument (Thermo Fisher Scientific, Inc.)

### Wound healing assay

The migration was identified by wound healing assay. Transfected cells were seeded in individual 6−well dishes and incubated at 37°C until reaching about 80–90% confluence. Then, the two cells were scratched with a constant-diameter stripe from the bottom of the wells by a sterile 200-ul pipette tip. Filming was performed at 0 and 24 h after wounding. A total of 10 areas were randomly selected to mark and measure.

### Transwell assay

The transfected LUAD cell suspensions (200 ml) were moved to the upper chamber of the transwell module (Corning, Inc.) and incubated for 24 h at 37°C and 5% CO_2_. The cells would invade the bottom chamber that contained the prepared medium (with 10% fetal bovine serum added). The invaded NCI-H2009 and A549 cells that existed in the lower chamber were thereby treated with methanol and 0.1% crystal violet. The cell invasion rate was measured by eluting the crystal violet which existed in the transwell by 33% acetic acid. Finally, we measured the OD 570 nm value in the eluted liquid.

### Dual−luciferase assay

The online tool TargetScan was applied to identify the potential binding sites. The wild-type site (wt) and mutant site (mut) sequences of MIR31HG (MIR31HG wt and MIR31HG mut) and TNFRSF21 (TNFRSF21 wt and TNFRSF21 mut), including the homologous binding sites of miR-193a-3p, were amplified and uniformly plugged in the vector pGL3 (Promega, Madison, WI, USA). Then, miR-193a-3p mimics were co−transfected with MIR31HG wt, MIR31HG mt, TNFRSF21 wt, or TNFRSF21 mut using Lipo3000. After 48 h, Dual−Luciferase Reporter Assay System provided from Promega was used to detect the luciferase activity.

### RNA immunoprecipitation assay

EZ−Magna RIP™ RNA−Binding Protein Immunoprecipitation Kit provided from Labbiotech was applied to execute the RNA immunoprecipitation (RIP) assay. NCI-H2009 and A549 were uniformly mixed with RIP buffer owning beads stuck by anti-Ago2 or anti-IgG (negative control) and incubated overnight. Finally, the obtained immunoprecipitated complexes were measured by real−time PCR.

### Western blot

RIP assay lysis buffer (Beyotime, Shanghai, China) was used to extract the protein from LUAD cells or tissues. After measuring the density of all proteins, the target proteins (30 µg/lane) were separated by SDS-PAGE (10%) and then carefully transferred onto polyvinylidene fluoride membranes (Bio-Rad, Hercules, CA, USA). Later on, the transferred membranes were blocked with silk milk (5%) for 1 h at 37°C and incubated with the primary anti-TNFRSF21 (catalogue no. ab8417; 1:800; Abcam; USA) and β-actin (catalog no. ab8226; 1:3,000; Abcam; USA) overnight at 4°C. On the second day, the membranes were then treated with a corresponding secondary antibody (catalog no. ab6721; 1:5,000; catalog no. ab6728; 1:5,000; Abcam; USA). Finally, the protein signals on the membrane were visualized by enhanced ECL detection kit (Beyotime, Shanghai, China).

### RT−qPCR analysis

Trizol (Beyotime, Shanghai, China) was employed to extract the total RNA from LUAD cells and tissues. RNA was reverse-transcribed into complementary DNA (cDNA) using SuperScript VILO cDNA Kit (Thermo Fisher Scientific, Inc.). SYBR Green qPCR Master Mix (Applied Biosystems, USA) was applied to detect the quantitative PCR from the 2^−ΔΔCq^ method. The primers are listed in **Table 1**.

**Table 1:** Primer list.

Gene	Primers
MIR31HG	Forward: 5′-TCCCAGTTTCAGACCACC-3′ Reverse: 5′-CCAGGCTATGTCTTTCCTCTAT-3′
TNFRSF21	Forward: 5′-ATTCCCCAGGCTGAGGACAAAC-3′ Reverse: 5′-ACACACACACACACCCCAAAC-3′
GADPH	Forward: 5′-ACCACAGTCCATGCCATCAC-3′ Reverse: 5′-TCCACCACCCTGTTGCTGTA-3′
U6	Forward: 5′-CTCGCTTCGGCAGCACA-3′ Reverse: 5′-AACGCTTCACGAATTTGCGT-3′
miR-193a-3p	Forward: 5′-CGCGAACTGGCCTACAAAGTG-3′ Reverse: 5′-AGTGCAGGGTCCGAGGTATT-3′

### Statistical analysis

One-way analysis of variance (ANOVA) and paired samples *t*-test were used to assess differences between groups. Pearson’s correlation test analyzed the correlations. SPSS 25.0 software and GraphPad Prism 8.0.1 were performed for statistical analyses. All experiments were performed independently and repeated three times. *P* < 0.05 was considered statistically significant.

## Results

### Identification of the expression of cuproptosis-related genes in LUAD

**Figure 1:**
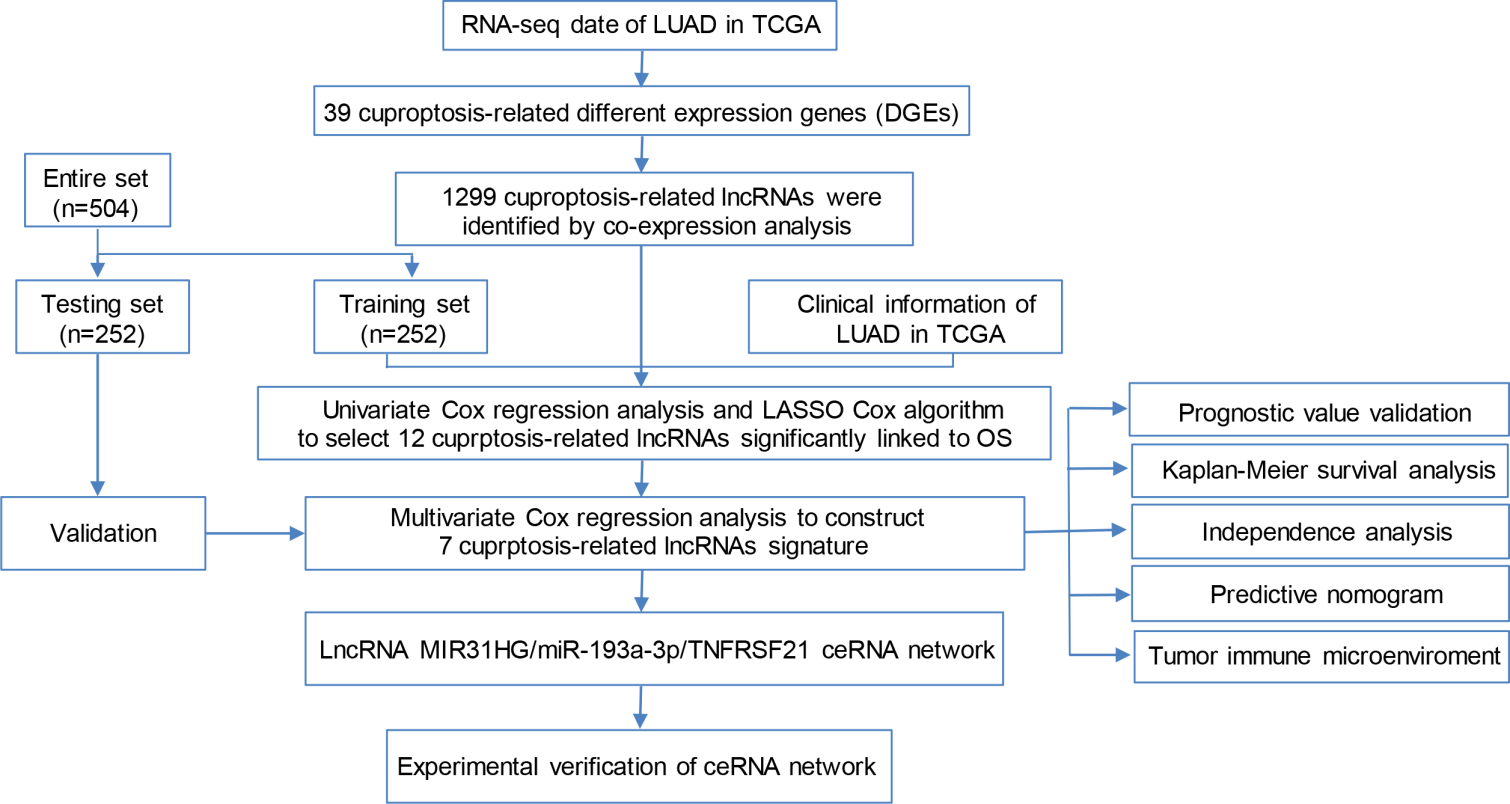
Flow chart of the study.

**Figure 2:**
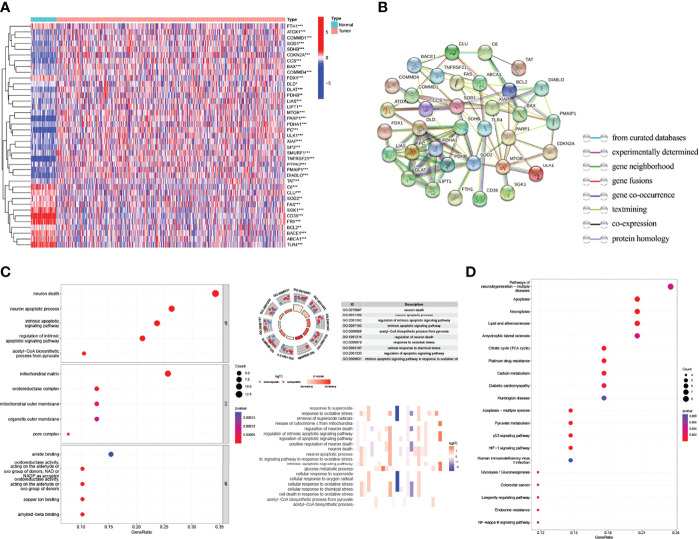
Identification of the expression and functional enrichment of cuproptosis-related genes in lung adenocarcinoma (LUAD). **(A)** Heat map reflecting the distribution of the 39 cuproptosis-related genes in LUAD and normal tissues. Red: upregulation; green: downregulation. **(B)** PPI network showing the interaction among 39 cuproptosis-related genes. **(C)** GO enrichment of cuproptosis-related genes. **(D)** Enriched KEGG pathways of cuproptosis-related genes. PPI, protein–protein interaction network; GO, Gene Ontology; KEGG, Kyoto Encyclopedia of Genes and Genomes. *p < 0.05; **p < 0.01; ***p < 0.001.

The flow chart of the study is shown in **Figure 1**. The expression degrees of 49 genes linked to cell cuproptosis were compared in LUAD and normal tissues from the TCGA dataset, and 39 cuproptosis-related genes were identified as DEGs. Then, 26 genes (CLU, PDHB, BCL2, COMMD1, *etc*.) were detected to be enriched, while 13 genes (CD36, TLR4, TNFRSF21, ABCA1, *etc*.) were decreased in the LUAD group relative to normal tissues (**Figure 2A**). PPI showed the interaction among 39 DEGs (**Figure 2B**).

### Biological functional enrichment research of cuproptosis-related DEGs

GO and KEGG databases were used to analyze the potential and meaningful function of 39 DEGs. The GO analysis suggested that these genes in the biological processes were enriched in “neuron death” and “neuron apoptotic process”. These genes in cell component were enriched in “mitochondrial matrix” and “oxidoreductase complex”. Alterations in molecular function were brimming with “amide binding” and “oxidoreductase activity” (**Figure 2C**). Moreover, the KEGG analysis indicated that these DEGs were enriched in “pathways of neurodegeneration multiple diseases”, “apoptosis”, “necroptosis”, and “p53 signaling pathway” (**Figure 2D**).

### Identification of cuproptosis-related lncRNAs and co-expression network construction

**Figure 3:**
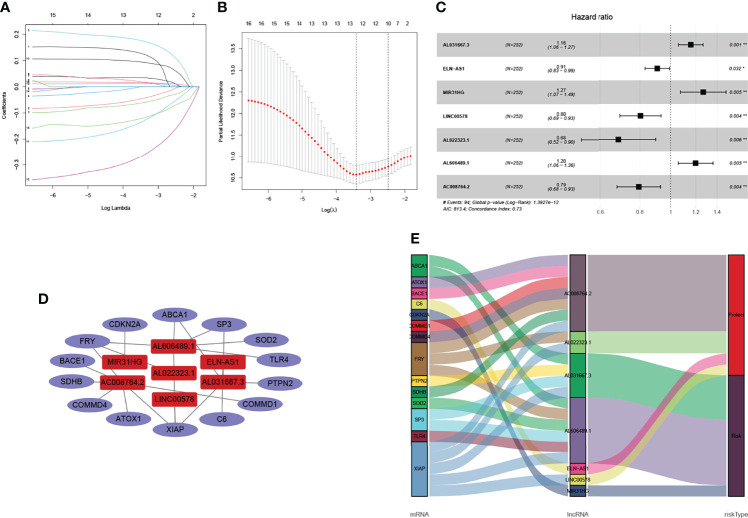
Identification of cuproptosis-related lncRNAs and co-expression network construction. **(A, B)** LASSO Cox algorithm was employed to establish a prognosis model. The color depth of the nodes depicted the corrected *P*-value of ontologies. The size of the nodes depicted the number of genes that are engaged in the ontologies. **(C)** A total of 7 lncRNAs were selected through subsequent multivariate analysis to construct the risk model. **(D)** Co-expression structure between cuproptosis-related lncRNAs and genes. **(E)** Sankey diagram was used to visualize the co-expression network.

Firstly, this work classified the included cases (*n* = 504) into training (*n* = 252) and validation (*n* = 252) cohorts at a 1:1 ratio. The clinicopathologic characteristics of LUAD patients are listed in **Table 2**. A total of 1,299 lncRNAs related to 39 DEGs were screened out for future analysis according to Pearson correlation method. Secondly, we operated the univariate Cox regression analysis and LASSO Cox algorithm to reduce multicollinearity, and we found 12 cuproptosis-related lncRNAs (**Figures 3A, B**). Finally, 7 lncRNAs, including AL031667.3, ELN-AS1, LINC00578, AL022323.1, AL606489.1, AC008764.2, and MIR31HG, were selected through subsequent multivariate analysis to construct the risk model, and the global *p*-value was 1.3927e-12 (**Figure 3C**). The co-expression network between cuproptosis-related lncRNAs and genes were constructed in **Figure 3D**. Among these 7 cuproptosis-related lncRNAs, AC008764.2, AL022323.1, ELN-AS1, and LINC00578 were identified as protective lncRNAs, while AL031667.3, AL606489.1, and MIR31HG were identified as risk lncRNAs (**Figure 3E**).

**Table 2:** The clinicopathologic characteristics of 504 lung adenocarcinoma patients in The Cancer Genome Atlas.

Characteristics	Training cohort (*n* = 252)	Validation cohort (*n* = 252)	Entire set (*n* = 504)
Age			
≤65 >65	134 (53.17%) 118 (46.83%)	122 (48.41%) 130 (51.59%)	256 (50.79%) 248 (49.21%)
Gender			
Female Male	131 (51.98%) 121 (48.02%)	139 (55.16%) 113 (44.84%)	270 (53.57%) 234 (46.43%)
T			
T1–T2 T3–T4 Unknown	214 (84.92%) 37 (14.68%) 1 (0.40%)	223 (88.49%) 27 (10.71%) 2 (0.80%)	437 (86.71%) 64 (12.70%) 3 (0.59%)
N			
N0 N1–N3 Unknown	166 (65.87%) 80 (31.75%) 6 (2.38%)	159 (63.10%) 88 (34.92%) 5 (1.98%)	325 (64.48%) 168 (33.33%) 11 (2.18%)
M			
M0 M1 Unknown	170 (67.46%) 12 (4.76%) 70 (27.78%)	167 (66.27%) 14 (5.56%) 71 (28.17%)	337 (66.87%) 26 (5.16%) 141 (27.98%)
Stage			
Stage I–stage II Stage III–stage IV	202 (80.16%) 50 (19.84%)	193 (76.59%) 59 (23.41%)	395 (78.37%) 109 (21.63%)

### Construction of a predictive risk model in LUAD patients

**Figure 4:**
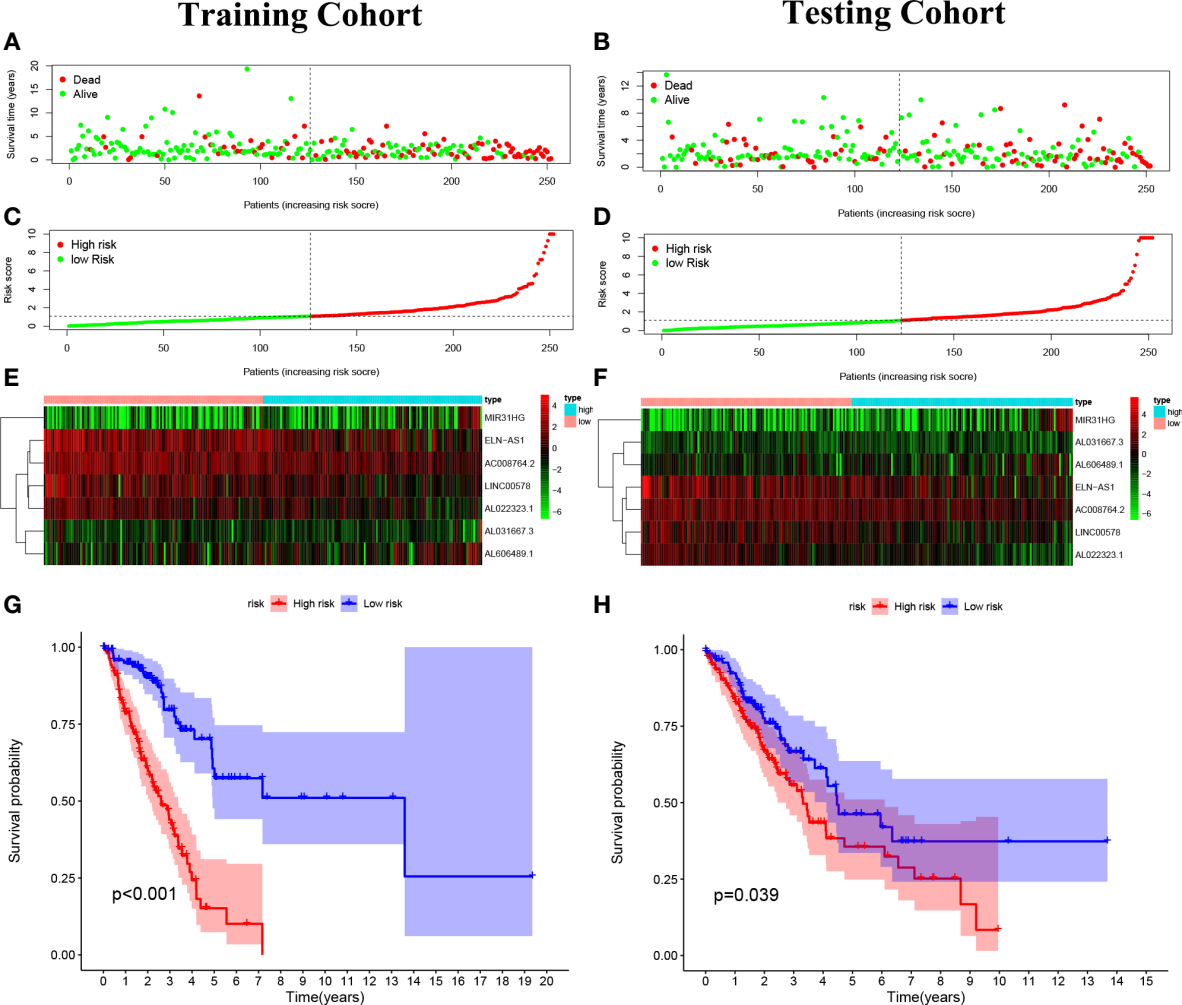
Construction of the predictive risk model in lung adenocarcinoma (LUAD) patients. **(A, B)** The risk score was calculated by 7 cuproptosis-related lncRNAs in the two cohorts (training and validation), and two risk groups were formed in LUAD patients. Green, low risk; red, high risk. **(C, D)** Survival status of LUAD patients. Green: survival; red: death. **(E, F)** The heat map indicates the expression degrees of 7 cuproptosis-related lncRNAs. **(G, H)** The Kaplan–Meier analysis revealed the overall survival in the two risk groups.

The 7 lncRNAs were identified in the risk model with “risk score” = AL022323.1 × (-0.379446) + AC008764.2 × (-0.231185) + LINC00578 × (-0.218454) + ELN-AS1 × (-0.097027) + AL031667.3 × (0.145940) + AL606489.1 × 0.180937 + MIR31HG × (0.235981). The LUAD patients in both training (*n* = 252) and testing (*n* = 252) cohorts were divided into high- and low-risk groups based on the median risk score (**Figures 4A–D**). The expression degrees of 7 lncRNAs in the two different groups were exhibited by a heat map (**Figures 4E, F**). Interestingly, patients with high risk had worse overall survival (OS) compared with patients with low risk as revealed by Kaplan–Meier analysis (**Figures 4G, H**).

### Prognosis value of model lncRNAs in LUAD

**Figure 5:**
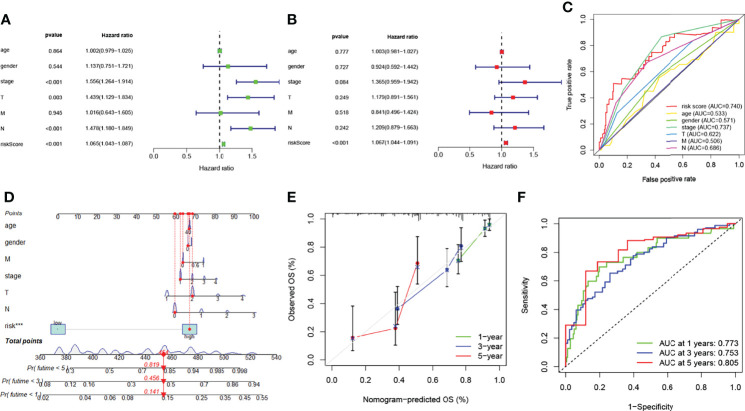
Prognosis value of model lncRNAs in lung adenocarcinoma (LUAD). **(A)** Univariate analysis of various clinical features and risk score. **(B)** Multivariate analysis of various clinical features and risk score. **(C)** ROC curves of the risk model (risk score: AUC = 0.740). **(D)** A nomogram was performed to predict the 1-, 3-, and 5-year survival. **(E)** Calibration curve of the nomogram model. **(F)** The results of ROC curves in predicting the LUAD survival rates. ROC, receiver operator characteristic; AUC, area under the curve.

Cox regression analyses pointed out that the score calculated by the corresponding model was an independent factor to predict the OS in LUAD relative to other clinical factors (**Figures 5A, B**). Meanwhile, the outcome of the ROC curve analysis suggested that the risk score tended to show more sensitivity and specificity than the other clinical features (risk score: AUC = 0.740) (**Figure 5C**).

### Construction and detection of the predictive nomogram in LUAD

The nomogram models were established to validate the LUAD patients’ survival value of 1, 3, and 5 years (**Figure 5D**). Risk score was identified as independent prognostic factor (***p < 0.001). The results of the calibration curve revealed that the nomogram model showed a significant accuracy in predicting the LUAD patients’ OS (**Figure 5E**). The model likewise showed high sensitivity and efficacy (1-year AUC = 0.773, 3-year AUC = 0.753, and 5-year AUC = 0.805) (**Figure 5F**). In short, these results identified that both risk model and nomogram model revealed that the overall survival rate can be predicted relatively well.

### Correlation analysis of different groups and tumor microenvironment infiltration

**Figure 6:**
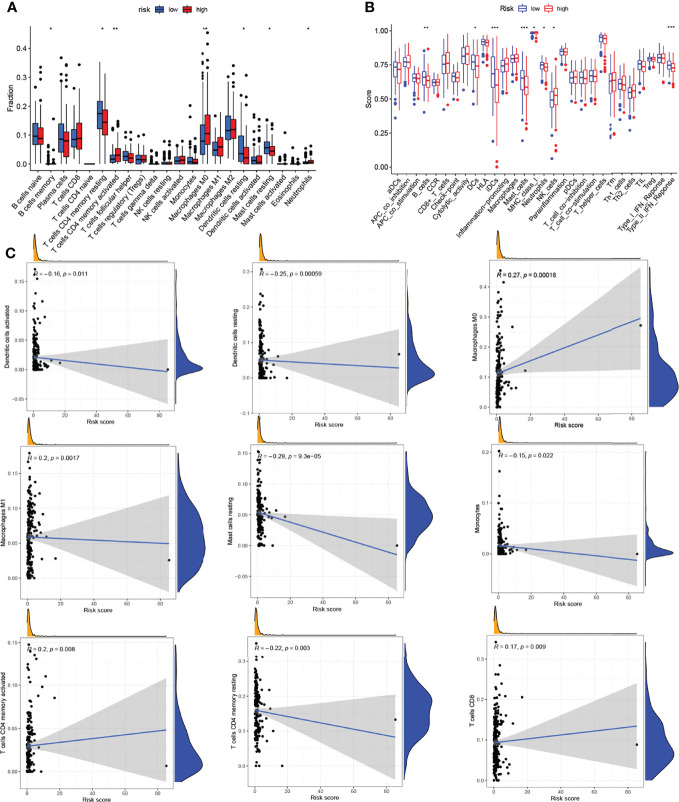
Analysis of the immune activity in different groups. **(A)** Comparison of various types of immune cells. **(B)** Comparison of various immune-correlated pathways. **(C)** Relationship between the infiltration of immune cells and the risk model. ^*^ *p* < 0.05; ^**^ *p* < 0.01; ^***^ *p* < 0.001.

To evaluate the role of the risk model in the immune microenvironment of LUAD, the CIBERSORT algorithm was performed to compare 22 different immune cell types in LUAD, finding that 7 of these immune cell types were differentially expressed in two risk groups (*p < 0.05 and **p < 0.01) (**Figure 6A**). Meanwhile, we discovered that, in the TCGA project, the low-risk group of the TCGA cohort had a significantly higher score of most immune-correlated pathways than the high-risk group (**Figure 6B**). In addition, the relationship between the risk model and infiltration of immune cells is described in **Figure 6C**, suggesting that there was a positive correlation between the survival outcome of LUAD patients and the high degrees of M0 macrophages, M1 macrophages, CD4 memory-activated T cells, and CD8 T cells, while there was a negative correlation between the survival outcome of LUAD patients and the high degrees of activated dendritic cells, resting dendritic cells, resting mast cells, monocytes, and resting CD4 memory T cells. To sum up, these results highlight the immunomodulatory effects of the risk model.

### Construction of the lncRNA MIR31HG/miR-193a-3p/TNFRSF21 regulatory axis

**Figure 7:**
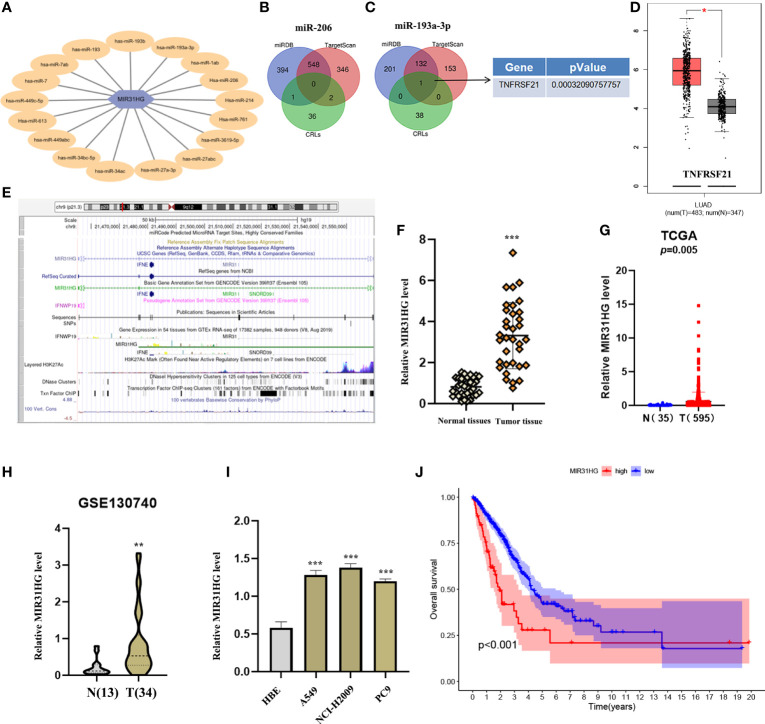
Construction of a regulatory axis of lncRNA–miRNA–mRNA. **(A)** lncRNA MIR31HG bound to 17 miRNAs. **(B, C)** Venn diagram identifying the downstream targets in miR-206 and miR-193a-3p, respectively, from miRDB and TargetScan databases. **(D)** The expression of TNFRSF21 was investigated by GEPIA database. **(E)** Lots of H3K27Ac marks existed in the promoter region of MIR31HG from the UCSC web server. **(F, G)** MIR31HG expression in lung adenocarcinoma (LUAD) specimens relative to normal tissues as detected by qRT-PCR and The Cancer Genome Atlas cohort. **(H)** MIR31HG expression in LUAD specimens relative to normal tissues as detected by GSE130740 cohort. **(I)** MIR31HG expression in different LUAD cell lines (A549, NCI-H2009, and PC9) compared with bronchial epithelioid cell (HBE) was estimated by qRT-PCR. **(J)** The overall survival time of patients with LUAD was measured by Kaplan–Meier analysis. ^*^ *p* < 0.05; ^**^ *p* < 0.01; ^***^ *p* < 0.001.

To further understand the potential mechanism of cuproptosis-related lncRNAs in LUAD, we constructed the network of lncRNA–miRNA–mRNA interaction regulatory axis. According to Mircode database, we found that lncRNA MIR31HG bound miRNAs using “Perl’” software (**Figure 7A**). Among these miRNAs, 2 miRNAs (miR-206 and miR-193a-3p) were identified to be less expressed in lung cancer (16, 17), which was contrary to the expression of the target lncRNA MIR31HG. Based on this result, we then explored its downstream mRNA targets to construct the miRNA–mRNA axis. According to the miRDB and TargetScan databases, cuproptosis-related mRNA (TNFRSF21) was identified as the downstream target of miR-193a-3p (**Figures 7B, C**). We then found that only TNFRSF21 had an upregulated expression in LUAD tissues according to the GEPIA (http://gepia.cancer-pku.cn/) database (**Figure 7D**).

### lncRNA MIR31HG is overexpressed in LUAD tissues and cell lines

**Figure 7E** shows that lots of H3K27Ac marks existed in the promoter region of lncRNA MIR31HG from the UCSC web server. To understand the role of lncRNA MIR31HG in LUAD, qRT-PCR analysis, TCGA database, and GEO dataset (GSE:130740) were used and identified the high level of MIR31HG in LUAD specimens relative to normal tissues (**Figures 7F–H**). We likewise selected several lung cancer cell lines (A549, NCI-H2009, and PC9) for experimental validation *in vitro*, with bronchial epithelioid cell (HBE) as the control group, and the result suggested that the expression of MIR31HG was also enhanced in lung cancer cell lines compared with the control group (**Figure 7I**). Additionally, we identified that LUAD patients with a higher expression level of lncRNA MIR31HG had a shorter OS time than those with a lower MIR31HG expression level (**Figure 7J**).

### lncRNA MIR31HG mainly locate in the cytoplasm and its knockdown inhibits LUAD cell proliferation, migration, and invasion

**Figure 8:**
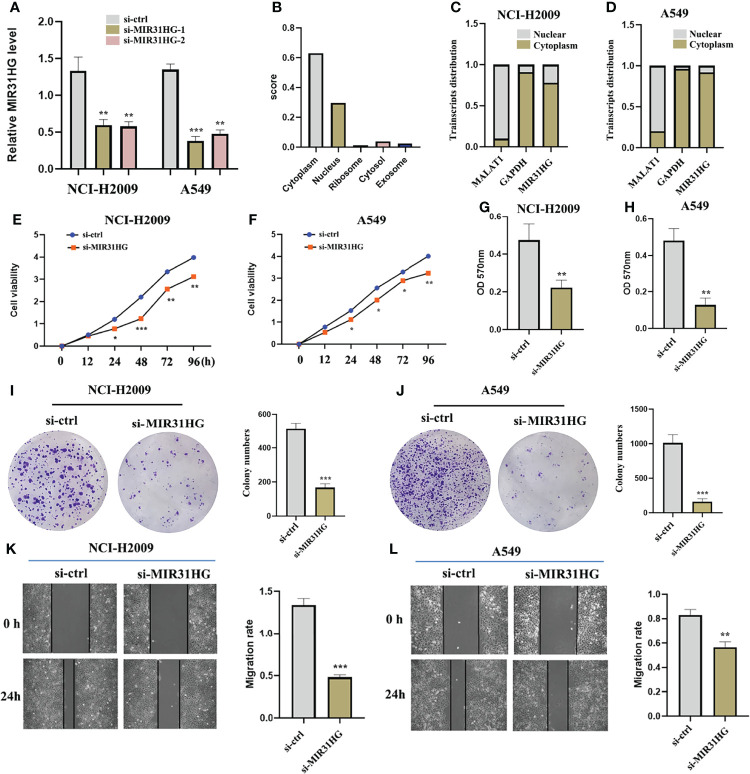
The effect of lncRNA MIR31HG on lung adenocarcinoma LUAD cell viability, migration, and invasion. **(A)** The efficiency of MIR31HG knockdown (si-MIR31HG-1 and si-MIR31HG-2) was assessed by qRT-PCR. **(B)** The subcellular localization of MIR31HG was predicted by “LncLocator”. **(C, D)** The relative lncRNA MIR31HG expression level both in the cytoplasm and the nucleus of the NCI-H2009 and A549 cell lines were simultaneously measured by qRT-PCR. **(E, F)** The proliferation of NCI-H2009 and A549 cells was detected by CCK-8 assays. **(G, H)** The invasion of NCI-H2009 and A549 cells was investigated by transwell assays. **(I, J)** The clone capacity of NCI-H2009 and A549 cells was identified by colony formation assay. **(K, L)** The migration of NCI-H2009 and A549 cells was determined by wound healing assays. ^*^ *p* < 0.05; ^**^ *p* < 0.01; ^***^ *p* < 0.001.

The subcellular localization analysis of lncRNA plays an essential role in exploring the functional mechanism of lncRNA (18). Based on this, we adopted a one-step method to completely isolate cytoplasmic RNA and nuclear RNA. The reverse transcription and assay analysis of lncRNA MIR31HG transcript levels by qRT-PCR revealed that the transcripts of lncRNA MIR31HG were mainly distributed in the cytoplasm of NCI-H2009 and A549 cells, which was consistent with the predicted result of “LncLocator” (http://www.csbio.sjtu.edu.cn/bioinf/lncLocator/) (**Figures 8B–D**). Subsequently, in order to determine whether lncRNA MIR31HG is involved in the initiation and progression of LUAD, functional interference techniques were used to evaluate the behavioral effects of lncRNA MIR31HG deletion. **Figure 8A** shows that the transfection was clearly successful in LUAD cell lines. The results of CCK-8 detection suggested that lncRNA MIR31HG interference could significantly inhibit the proliferation activity of NCI-H2009 and A549 cells (**Figures 8E, F**). Similarly, the colony formation assays revealed that the clone capacity of LUAD cells was remarkably inhibited by the silencing of lncRNA MIR31HG (**Figures 8I, J**). The data from wound healing and transwell analyses showed that NCI-H2009 and A549 cell lines exhibited significantly attenuated migration and invasion functions after interference with MIR31HG (**Figures 8G, H, K, L**). These findings demonstrate that lncRNA MIR31HG played a key role in stimulating the progression of LUAD.

### miR-193a-3p is sponged by lncRNA MIR31HG

**Figure 9:**
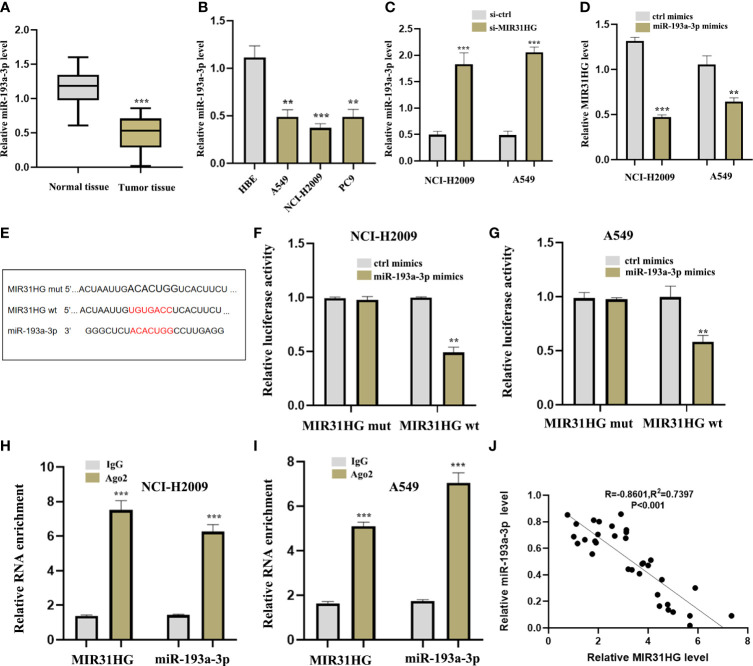
lncRNA MIR31HG acted as ceRNA for miR-193a-3p. **(A)** miR-193a-3p expression in lung adenocarcinoma (LUAD) specimens compared with normal tissues as detected by qRT-PCR. ^***^ *p* < 0.001 *vs*. normal tissue. **(B)** miR-193a-3p expression in different LUAD cell lines (A549, NCI-H2009, and PC9) relative to bronchial epithelioid cell (HBE) was estimated by qRT-PCR. **(C)** miR-193a-3p expression following si-MIR31HG transfection was assessed by qRT-PCR. **(D)** MIR31HG expression following miR-193a-3p overexpression was measured by qRT-PCR. **(E)** Schematic diagram of the predicted interacting sites. **(F, G)** The relationship between MIR31HG and miR-193a-3p in NCI-H2009 and A549 cells was performed by dual-luciferase reporter assay. **(H, I)** The immunoprecipitation of MIR31HG and miR-193a-3p in NCI-H2009 and A549 cells was determined by RNA immunoprecipitation experiment. **(J)** The relationship between MIR31HG and miR-193a-3p was investigated by Pearson’s analysis. ^**^ *p* < 0.01; ^***^ *p* < 0.001.

To further explore the specific mechanism of lncRNA MIR31HG as ceRNA in LUAD, we identified the target miR-193a-3p by Mircode database (**Figure 7A**). In addition, we noticed that the expression level of miR-193a-3p was low in both LUAD tumor tissues and cell lines compared with normal groups, respectively (**Figures 9A, B**). qRT-PCR was investigated following si-MIR31HG or miR-193a-3p mimic transfection, revealing that silencing of lncRNA MIR31HG significantly promoted the expression of miR-193a-3p relative to “si-ctrl” (**Figure 9C**), whereas miR-193a-3p over-expression clearly decreased MIR31HG expression (**Figure 9D**). **Figure 9E** shows some binding sites between miR-193a-3p and MIR31HG 3′UTR. The luciferase assay clearly verified the potential relationship between lncRNA MIR31HG and miR-193a-3p (**Figures 9F, G**). The RIP analysis suggested that lncRNA MIR31HG and miR-193a-3p had obvious immunoprecipitation in Ago2 complex in LUAD cell lines NCI-H2009 and A549 (**Figures 9H, I**). In addition, we further found that there existed a negative correlation between lncRNA MIR31HG and miR-193a-3p (**Figure 9J**).

### Interference of miR-193a-3p reverses the effect of lncRNA MIR31HG on LUAD cells

**Figure 10:**
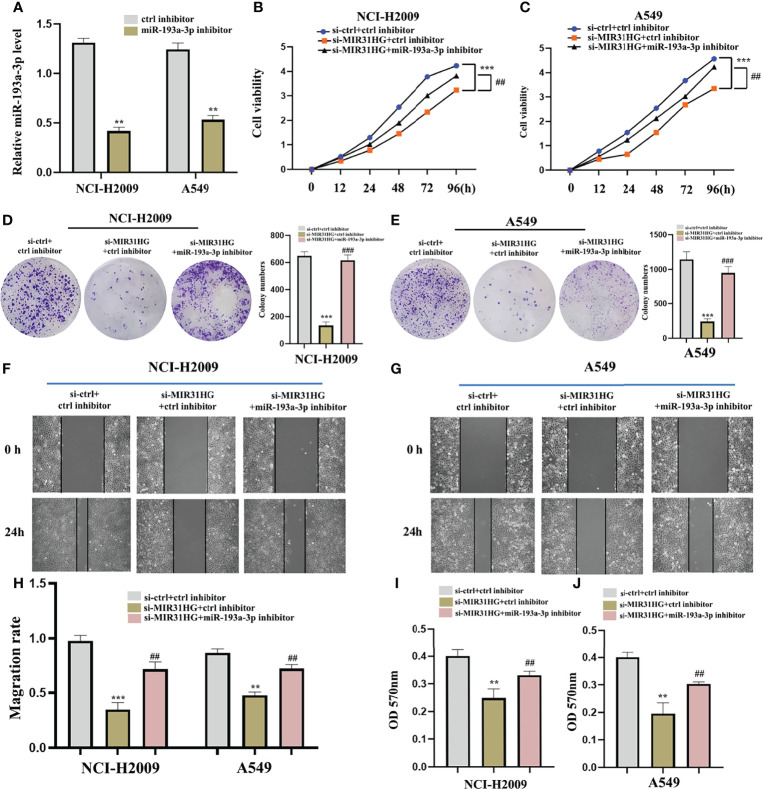
miR-193a-3p reversed the lncRNA MIR31HG knockdown effects on lung adenocarcinoma cells. **(A)** The expression of miR-193a-3p was investigated by qRT-PCR. **(B, C)** The proliferation of NCI-H2009 and A549 cells were detected by CCK-8 assays. **(D, E)** The clone capacity of NCI-H2009 and A549 cells was identified by colony formation assay. **(F-H)** The migration of NCI-H2009 and A549 cells was determined by wound healing assays. **(I, J)** The invasion of NCI-H2009 and A549 cells was investigated by transwell assays. ^**^ *p* < 0.01; ^***^ *p* < 0.001 *vs*. ctrl inhibitor, ^##^ *p* < 0.01; ^###^ *p* < 0.01 *vs*. si-MIR31HG+miR-193a-3p inhibitor.

To further ascertain whether the effect of lncRNA MIR31HG on LUAD cells is affected by miR-193a-3p, we co-transfected miR-193a-3p inhibitor with si-MIR31HG. **Figure 10A** shows that the interference effect of miR-193a-3p is successful. The proliferation activity of LUAD cell lines NCI-H2009 and A549 was blocked by si-MIR31HG and rescued by the addition of miR-193a-3p inhibitor (**Figures 10B, C**). Similarly, colony formation assays established that the clone capacity of LUAD cells was remarkably inhibited by si-MIR31HG but returned by adding miR-193a-3p inhibitor (**Figures 10D, E**). Moreover, the ability of cell migration and invasion was simultaneously decreased by si-MIR31HG but restored by transfecting miR-193a-3p inhibitor (**Figures 10F–J**). These findings proved that the MIR31HG interference inhibited the malignant activities of LUAD cells *via* upregulating miR-193a-3p.

### miR-193a-3p targets downstream TNFRSF21

**Figure 11:**
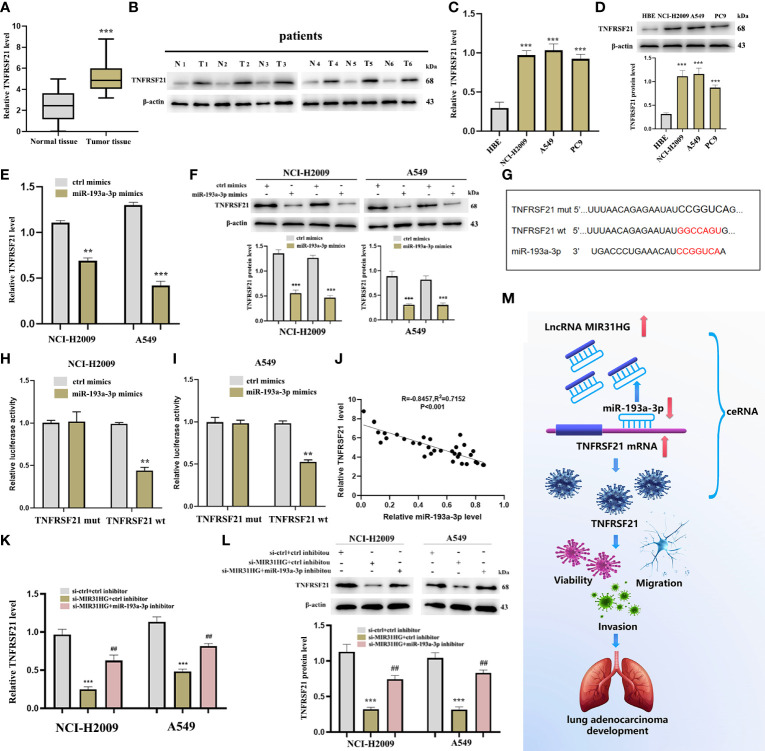
TNFRSF21 was a downstream target of miR-193a-3 and lncRNA MIR31HG sponged miR-193a-3p to upregulate TNFRSF21. **(A)** TNFRSF21 expression in lung adenocarcinoma (LUAD) specimens compared with normal tissues as detected by qRT-PCR. ^***^ *p* < 0.001 *vs*. normal tissue. **(B)** TNFRSF21 expression in 6 LUAD patients relative to normal tissues as detected by western blot. **(C, D)** TNFRSF21 expression in different LUAD cell lines (A549, NCI-H2009, and PC9) relative to bronchial epithelioid cell (HBE) was estimated by qRT-PCR or western blot. ^***^ *p* < 0.001 *vs*. HBE. **(E, F)** TNFRSF21 expression following miR-193a-3p overexpression was assessed by qRT-PCR or western blot. ^**^ *p* < 0.01; ^***^ *p* < 0.001 *vs*. ctrl mimics. **(G)** Schematic diagram of the predicted interacting sites. **(H, I)** The relationship between TNFRSF21 and miR-193a-3p in NCI-H2009 and A549 cells was performed by dual-luciferase reporter assay. ^**^ *p* < 0.01 *vs*. ctrl mimics. **(J)** Relationship between TNFRSF21 and miR-193a-3p investigated by Pearson’s analysis. **(K, L)** TNFRSF21 expression following si-MIR31HG or si-MIR31HG + miR-193a-3p inhibitor was assessed by qRT-PCR or western blot. ^***^ *p* < 0.001 *vs*. ctrl inhibitor; ^##^ *p* < 0.01 *vs*. si-MIR31HG + miR-193a-3p inhibitor. **(M)** Schematic diagram of the mechanism of lncRNA MIR31HG/miR-193a-3/TNFRSF21 regulatory axis.

The cuproptosis-related mRNA (TNFRSF21) was identified as the downstream target of miR-193a-3p from miRDB and TargetScan databases (**Figure 7C**). Firstly, we noticed that TNFRSF21 was clearly upregulated in both LUAD tumor tissues and NCI-H2009 and A549 cells compared with normal groups, respectively (**Figures 11A–D**). In addition, the level of TNFRSF21 was effectively suppressed by the over-expression of miR-193a-3p (**Figures 11E, F**). **Figure 11G** shows some binding sites between miR-193a-3p and TNFRSF21 using the online tool TargetScan. The luciferase assay clearly verified the potential interacting sites between miR-193a-3p and TNFRSF21 (**Figures 11H, I**). We further found a negative correlation between miR-193a-3p and TNFRSF21 (**Figure 11J**).

### lncRNA MIR31HG sponges miR-193a-3p to upregulate TNFRSF21

Thereafter, we investigated whether lncRNA MIR31HG could sponge miR-193a-3p to upregulate TNFRSF21. The result of qRT-PCR identified that lncRNA MIR31HG inhibitor significantly inhibited the expression of TNFRSF21, but these alterations were reversed by miR-193a-3p inhibitor (**Figure 11K**), which was consistent with the outcome of the Western blot (**Figure 11L**). **Figure 11M** shows the schematic diagram of the mechanism of the lncRNA MIR31HG/miR-193a-3/TNFRSF21 regulatory axis.

## Discussion

Cuproptosis, as a new type of death, is receiving more and more attention. Our study systematically identified cuproptosis-related lncRNAs based on the involvement of lncRNAs and cuproptosis-related mRNAs in LUAD. After that, a unique risk model with various features was constructed to predict the prognosis of LUAD patients. Firstly, 39 differentially expressed cuproptosis-related genes were identified as “DEGs” from all cuproptosis-related genes by comparing LUAD and normal tissues. Both GO and KEGG databases indicated that these “DEGs” were enriched in the pathways of neurodegenerative diseases. In the nervous system, copper is involved in myelination, excitotoxic cell death, synaptic activity, and neurotrophic factor-induced signaling cascades (19). Aberrant copper homeostasis could lead to multiple pathological sequelae, including cancer, inflammation, and neurodegeneration. Correcting disturbed copper homeostasis is a promising therapeutic strategy for neurodegenerative diseases (20). Secondly, a risk model consisting of 7 lncRNAs was built to form two risk groups of LUAD patients. Thirdly, the risk score was proved to be an independent prognostic factor with a high degree of sensitivity and specificity compared with other clinical factors. Moreover, the analysis of the infiltration of immune cells and the risk model highlighted the immunomodulatory effects of cuproptosis-related lncRNAs in these two risk groups. Finally, we predicted and verified the network of lncRNA MIR31HG/miR-193a-3p/TNFRSF21, which may play a potential role in the progression of LUAD.

Multiple studies have confirmed that lncRNAs played important roles in different aspects of LUAD development—for example, Deng *et al*. reported that the lncRNA LINC00472 inhibits the migration and invasion of LUAD by regulating YBX1 (21). Qu *et al*. discovered that PD-L1 lncRNA splice isoform stimulated the progression of LUAD *via* the c-Myc axis (22). However, studies on cuproptosis-related lncRNAs in LUAD, especially their potential ability to predict prognosis in these LUAD patients, are fairly inadequate. Therefore, our work established a predictive model on the basis of 7 cuproptosis-related lncRNAs, including AL031667.3, ELN-AS1, LINC00578, AL022323.1, AL606489.1, AC008764.2, and MIR31HG. This means that, among these lncRNAs, some lncRNAs have been reported to be involved in the pathogenesis of several tumor diseases—for instance, Zheng *et al*. manifested that lncRNA AL031667.3 played a risk biomarker role in the prognosis of lung adenocarcinoma (23). Wang *et al*. detected that lncRNA ELN-AS1 was identified as a protective factor of endometrial cancer patients (24). Moreover, lncRNA LINC00578 could inhibit the tumor proliferation of pancreatic cancer (25) and lung adenocarcinoma (26). In addition, lncRNA AL022323.1 was identified as a protective factor of colorectal cancer with low aggression (27). Guo *et al*. revealed that lncRNA AL606489.1 was referred to as a prognostic marker and dangerous effector in lung adenocarcinoma (28). lncRNA MIR31HG has been reported to promote glioblastoma progression by regulating Wnt/β-catenin signaling (29). These reports likewise supported our conclusion that lncRNAs AL031667.3, AL606489.1, and MIR31HG were the dangerous factors of LUAD, while ELN-AS1, LINC00578, and AL022323.1, as protective factors, played an important role in prolonging LUAD patients’ survival time, which were consistent with what we found in this research.

Immunity therapy is closely related to the prognosis of cancer patients, and its positive response usually depends on the dynamic regulation between tumor cells and immune modulators in the tumor microenvironment (TME) (30). Therefore, effective exploration of the immunological characteristics of the TME will be conducive to a rapid discovery of a variety of new immunity therapy strategies and identification of more potential clinical prognostic biomarkers (31, 32). From the results of our study, immune-correlated infiltrating cells and immune-interrelated pathways were found to be mostly concentrated in the low-risk group, indicating that immune-suppressive therapy might be more effective in the low-risk group of LUAD patients.

A huge number of studies have proved that lncRNAs could sponge microRNA (miRNA) loci and regard as competing endogenous RNAs (ceRNAs), thus effecting and adjusting the biological activities of downstream mRNAs (33, 34). The lncRNA-correlated ceRNAs have been recently elucidated to play an irreplaceable role in the development of various cancers—for example, lncRNA-CDC6 promoted breast cancer progression by regulating the axis of microRNA-215/CDC6 (35). lncRNA HOXD-AS1 may, as a ceRNA, stimulate liver cancer metastasis (36). lncRNA MT1JP functioned as a ceRNA to regulate miR-92a-3p/FBXW7 in gastric cancer (37). These results discovered that the axis had been extensively explored and reported in many diseases. Therefore, the novel network of lncRNA MIR31HG/miR-193a-3p/TNFRSF21 in LUAD was constructed by using biological tools.

In non-small cell lung cancer, lncRNA MIR31HG could sponge miR-241 to upregulate SP1, thus stimulating tumor progression (38). Upregulation of lncRNA MIR31HG acted as an oncogene by stimulating the Wnt/β-catenin axis in lung cancer (39). In this work, we illustrated that lncRNA MIR31HG and TNFRSF21 were over-expressed, while miR-193a-3p was downregulated in human LUAD tumor tissues and cells. Moreover, functional experiments were executed using si-MIR31HG transfection, revealing that the proliferation, migration, and invasion of LUAD cells were inhibited following the interference of MIR31HG, which were consistent with previous reports on lncRNA MIR31HG. In addition, Liu *et al*. detected that miR-193a-3p expression was decreased and could act as a tumor suppressor in lung cancer (40). This report supported our experimental results on the low expression of miR-193a-3p in LUAD. In our study, we predicted that lncRNA MIR31HG might interact with miR-193a-3p according to Mircode database. The RIP analysis suggested that lncRNA MIR31HG could directly integrate with miR-193a-3p in the level of Ago2 complex, and we also found a negative correlation between lncRNA MIR31HG and miR-193a-3p in LUAD. Moreover, the knockdown of miR-193a-3p could partly weaken the effect of lncRNA MIR31HG interference on LUAD cells. These findings not only further defined the tumor suppressor properties of miR-193a-3p but also identified that miR-193a-3p was involved in the development of LUAD through the ceRNA regulatory pattern. Based on this result, we then explored miR-193a-3p′ downstream mRNA target. Finally, the cuproptosis-related mRNA (TNFRSF21) was identified as the downstream target of miR-193a-3p from miRDB and TargetScan databases. Our work found that TNFRSF21 showed a high expression level in NCI-H2009 and A549 cells compared with the normal group, which was supported by RT-qPCR and western blot. More interestingly, the regulation between lncRNA MIR31HG and TNFRSF21 was mediated by miR-193a-3p. In short, we concluded that lncRNA MIR31HG upregulated TNFRSF21 through sponging miR-193a-3p, which might be the indispensable mechanism of lncRNA MIR31HG-regulated LUAD progression.

Unfortunately, there are still many defects in our current research that need further improvement. First of all, the risk model in this study was mainly established from the TCGA LUAD cohort, so it is best to use the GEO cohort to further verify the accuracy of the LUAD patients’ prognosis. Secondly, there is a lack of analysis of the relationship between cuproptosis and lipid metabolism TCA. Moreover, we preliminarily predicted and verified that the network of lncRNA MIR31HG/miR-193a-3p/TNFRSF21 might play a potential role in LUAD *in vitro*, but more *in vivo* tests are still needed for deep verification, which is also the focus of our future work.

## Conclusion

To summarize, we successfully identified 7 cuproptosis-related lncRNAs—AL031667.3, ELN-AS1, LINC00578, AL022323.1, AL606489.1, AC008764.2, and MIR31HG. On the basis of these lncRNAs, a valid predictive model was established for LUAD patients’ clinical prognosis, which proved to be an effective independent factor compared with other clinical features. The correlation between cuproptosis-related lncRNAs and immune infiltration was elucidated based on the overall risk score of groups, which would lay a foundation to improve anti-tumor immunity and develop a new treatment system for LUAD. Interestingly, our research also predicted and verified the network of lncRNA MIR31HG/miR-193a-3p/TNFRSF21. We revealed the oncogenic function of lncRNA MIR31HG in the malignant progression of LUAD and remarkably identified its potential mechanism by regulating the miR-193a-3p/TNFRSF21 axis, which might be beneficial to further elucidate the pathogenesis of LUAD and provide new ideas for clinical treatment.

## Data availability statement

The original contributions presented in the study are included in the article/supplementary material. Further inquiries can be directed to the corresponding author.

## Ethics statement

The studies involving human participants were reviewed and approved by the Ethics Committee of The First Affiliated Hospital of Jinan University. The patients/participants provided their written informed consent to participate in this study.

## Author contributions

XM, DH, and MX conceived and designed the experiments. XM and DH conducted the research. XM, DH, PY, MX, and YL contributed materials and analysis tools. XM, DH, PY, MX, AN, FM, SB, and GJ analyzed the results. XM and DH wrote the paper. All authors contributed to the article and approved the submitted version.

## Funding

This study was supported by the National Natural Science Foundation of China (no. 81774376) and the Science and Technology Foundation of Guangzhou (no. 201803010059).

## Conflict of interest

The authors declare that the research was conducted in the absence of any commercial or financial relationships that could be construed as a potential conflict of interest.

## Publisher’s note

All claims expressed in this article are solely those of the authors and do not necessarily represent those of their affiliated organizations, or those of the publisher, the editors and the reviewers. Any product that may be evaluated in this article, or claim that may be made by its manufacturer, is not guaranteed or endorsed by the publisher.
